# Prevalence and chemotherapy of *Staphylococcus aureus* mastitis in dairy cattle

**DOI:** 10.1371/journal.pone.0315480

**Published:** 2025-02-13

**Authors:** Asjad Umair Shah, Jawaria Ali Khan, Muhammad Avais, Syed Haider Zaman, Zubir Munir, Safdar Abbas, Mahnoor Tariq, Muneeb ur Rahman, Fiza Tariq, Saqib Nawaz, Dalia Fouad, Aftab Ahmad Anjum, Qazi Israr Ahmed

**Affiliations:** 1 Department of Veterinary Medicine, Faculty of Veterinary Sciences, University of Veterinary and Animal Sciences, Lahore, Pakistan; 2 Livestock and Dairy Development Department, Government of Punjab, Lahore, Pakistan; 3 Livestock and Dairy Development (Extension), Khyber Pakhtunkhwa, Peshawar, Pakistan; 4 Shanghai Veterinary Research Institute, The Chinese Academy of Agricultural Sciences (CAAS), Shanghai, People’s Republic of China; 5 Department of Zoology, College of Science, King Saud University, Riyadh, Saudi Arabia; 6 Institute of Microbiology, Faculty of Veterinary Sciences, University of Veterinary and Animal Sciences, Lahore, Pakistan; 7 Livestock and Dairy Development (Extension), Balochistan, Quetta, Pakistan; ICAR-National Research Center on Pig, INDIA

## Abstract

This study aimed to isolate and characterize *Staphylococcus aureus (S*. *aureus)* from samples of mastitis milk taken from dairy cattle in Punjab’s Narowal District. 200 milk samples were collected aseptically from different dairy herds in the district, including clinical mastitis (CM) and sub-clinical mastitis (SCM) cows. Blood agar and mannitol salt agar were used for *S*. *aureus* isolation and identification. Selective media were then used for additional purification. Observations of morphological and biochemical traits verified the existence of *S*. *aureus*. Through questionnaire-based surveys, the prevalence of *S*. *aureus* mastitis was identified, and risk variables linked to its incidence were evaluated. The findings showed that *S*. *aureus* mastitis was prevalent in 42.5% of cases, with sub-clinical cases having a greater incidence (45.8%) than clinical cases (37.5%). Risk factors such as distance from dung pile to stall(m), source of water, dung removal per day, lactation period (weeks), parity, effect of milk yield (L), traumatic injury of udder, housing type, floor type, teat dipping, and bedding type was identified and their relationship to the occurrence of *S*. *aureus* mastitis was studied. Tests for antibiotic sensitivity revealed that *S*. *aureus* isolates were responsive to gentamycin, ceftiofur, tetracycline, enrofloxacin, and ciprofloxacin but extremely resistant to penicillin and amoxicillin. Additionally, the Somatic Cell Count (SCC) and California Mastitis Test (CMT) were used at different intervals to assess the effectiveness of the medication. Furthermore, compared to other treatment groups, a larger percentage of cure rates was seen in the groups receiving ceftiofur and enrofloxacin. Overall, this study contributes to the development of more effective management methods for *S*. *aureus* mastitis in dairy calves by offering insightful information about the condition’s prevalence, risk factors, antibiotic sensitivity, and effectiveness of treatment.

## 1. Introduction

Mastitis, the most prevalent disease in cows, significantly impacts the global dairy industry and is driven by multiple factors leading to decreased milk quality and quantity, increased culling rates, and substantial economic losses. There are two types of mastitis: clinical mastitis (CM) and subclinical mastitis (SCM) [1]. CM varies from mild to severe, presenting symptoms such as pain, swelling, decreased milk secretion with abnormal characteristics, and fever, potentially with systemic illness. In contrast, SCM shows no visible signs or changes in milk color, quality, or quantity but is characterized by a significant increase in somatic cell count due to leukocyte influx. Compared to CM, SCM is more detrimental, accounting for 70–80% of total milk production losses and requiring extended treatment due to its persistent nature [2].

Mastitis is caused by a variety of pathogens and is categorized epidemiologically into two types: contagious and environmental [3]. Contagious pathogens are primarily found in the udders of infected cows, serving as the main reservoir. These pathogens are transmitted from cow to cow, mainly during milking, and typically lead to chronic subclinical infections with occasional clinical flare-ups. Common contagious pathogens include *Staphylococcus aureus*, *Streptococcus agalactiae*, *Mycoplasma spp*., and *Corynebacterium bovis* [4]. On the other hand, environmental mastitis refers to intra-mammary infections caused by pathogens whose primary reservoir is the cow’s environment. Environmental pathogens include *E*. *coli*, *Klebsiella spp*., *Streptococcus dysgalactiae*, and *Streptococcus uberis*. Infections caused by these pathogens are typically clinical in nature and of short duration [5].

Staphylococcus aureus is a common cause of subclinical mastitis. In Pakistan, the reported prevalence of *S*. *aureus* mastitis in dairy herds is 30.32% [6]. Globally, the prevalence varies, from 5.6% in Korea to 70% in Hungary, with rates of 36.23% in China and 46.6% to 62.4% in the United States. Numerous studies have reported antibiotic resistance genes among *S*. *aureus* isolates [7], highlighting the need for responsible antimicrobial use by clinicians and prioritizing the search for alternative therapies to reduce antimicrobial use (AMU) and the emergence of antimicrobial resistance (AMR) in dairy products, which is crucial for veterinary medicine and public health [8].

The presence of antibiotic residues in milk can impact consumer health by disrupting gut flora, causing allergic reactions, and reducing antibiotic effectiveness [9]. In Pakistan, many farmers cannot afford modern healthcare, resulting in poor livestock health and financial losses. As a cost-effective alternative, plant-based ethno-medicine is increasingly used in rural areas to treat mastitis and other illnesses [10].

The objective of this study was to determine the prevalence of clinical and sub-clinical mastitis, identify associated risk factors, isolation of *S*. *aureus*, a major causative agent of contagious mastitis, and evaluate its treatment efficacy.

## 2. Materials and methodology

### 2.1 Ethical statement

All protocols of this study were approved by the Ethical Committee (for the use and care of animals) of the University of Veterinary and Animal Sciences, Lahore, Pakistan (Letter No: DR/550, Dated 07-09-2022). The owner of the animals filled out a written Performa / consent form.

### 2.2 Data collection

Data on various mastitis risk factors believed to influence its occurrence in dairy herds was gathered using a structured questionnaire with close-ended questions. Factors included age, health condition, feed, water quality, milk physical characteristics, milking practices, pre- and post-milking procedures, and udder/teat lesions Farmers having daily manure cleaning routine and good drainage system on their farm were randomly selected to answer the Questionnaire. Through questionnaires, different risk factors and the prevalence of mastitis was determined according to the formula given by [11].


$$Prevalence\;\left(\%\right)=\frac{Number\;of\;mastitis\;positive\;animals}{Number\;of\;total\;animals\;examined}\;x\;100$$

### 2.3 Field diagnosis of mastitis

Each cow was clinically examined for signs related to the udder and teats, including any noticeable abnormalities such as inflammatory swelling, pain, fibrosis, lesions or visible injuries, tissue atrophy, and teat blindness. Milk samples were also assessed for changes in color, odor, and consistency, with indicators of clinical mastitis including the presence of clots, flakes, blood, and other consistency alterations, alongside morphological changes in the udder and teats. For the screening of subclinical mastitis, California Mastitis Test (CMT) was used and scored from 0 to 4 as described in Table 1.

**Table 1:** Scoring of California mastitis test and its interpretation.

Score	Gelling/Thickening	Interpretation
0	None	Negative
1	Very Mild	Traces
2	Mild	Weak positive
3	Moderate	Distinct positive
4	Heavy, almost solidifies	Strong positive

### 2.4 Collection and handling of milk sample

A total of 200 milk samples were collected from mastitic dairy cattle for isolation and identification of *S*. *aureus*. The milk samples of both clinical and sub-clinical mastitic animals were collected aseptically. The samples were collected from various dairy herds in the district of Narowal. For sampling every teat end was scrubbed vigorously by using a pledget of cotton moistened with 70% ethanol. One pledget was used only for one teat and made it possible to place the vial (Collection Tube) as horizontally as it was possible, without touching the inner surface; the lid of the vial was removed. About 5ml of milk was collected aseptically after discarding the first few streaks. Immediately after collection, samples were transported in an icebox (4–8°C) for bacteriological examination at the Animal Health Research Laboratory, Department of Veterinary Medicine, University of Veterinary and Animal Sciences, Lahore.

#### 2.4.1 California mastitis test (CMT)

**Fig 1:**
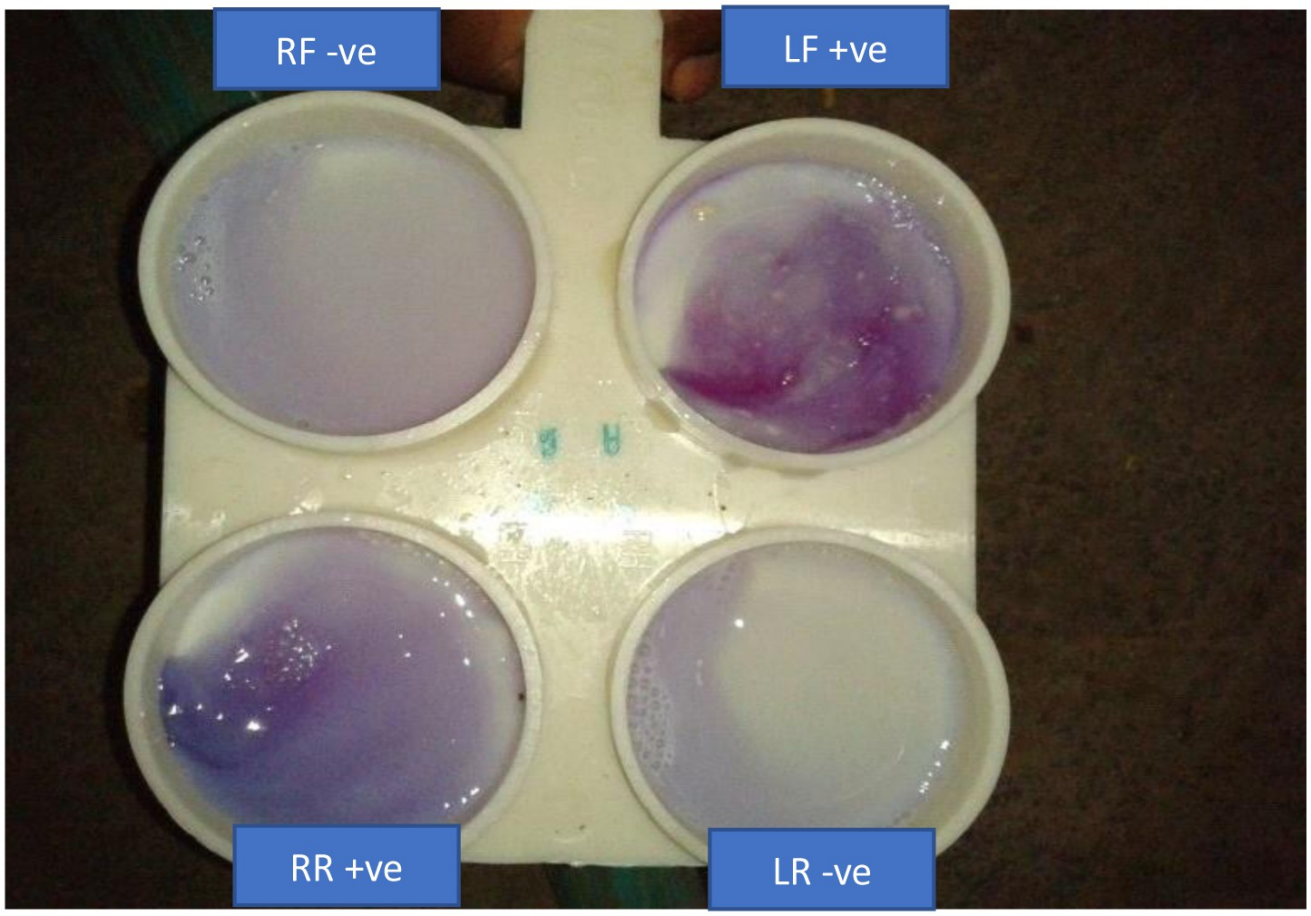
Interpretation of sample for CMT RF (Right Front), LF (left front), RR (Right Rear), LR (Left Rare).

This test was described by [12]. 2–3 ml of the milk sample was collected from each quarter of the animal onto a paddle (Fig 1). An equal amount of CMT reagent was added and swirled/shaken for about 10 seconds and the results were recorded between 10–20 seconds before disappearing of result. The resulting clot/gel formation was scored (Table 1) as described by [12]. A score of 0 was interpreted as negative, scores of 1, 2, and 3 indicated traces, weak positive and distinct positive results, respectively, suggesting subclinical mastitis, while a score of 4 as strongly positive (indicating clinical mastitis).

#### 2.4.2 Bacteriological examination

The procedure described by [13] for the culturing of milk samples was followed to identify mastitis causing pathogens. The samples of mastitic milk were shaken gently 8 times for a uniform dispersion of the pathogens and then streaked (0.01 ml of sample) on blood agar plates using a platinum loop for primary growth, and the plates were then incubated at 37 °C for 24 hours. After incubation, elevated opaque colonies with a diameter of 1–2 mm were assumed *Staphylococcus* positive. Furthur, these colonies were cultured on Mannitol Salt Agar (MSA) for the differentiation of *S*. *aureus* that produes yellow colored mannitol fermenting colonies. This was followed by the microscopic analysis using Gram’s staining showing irregular clusters of voilet colored cocci. Final confirmation for *S*. *aureus* was done through different biochemical tests.

### 2.5 Biochemical tests

#### 2.5.1 Catalase test

A catalase test was performed to check the production of catalase enzyme by Gram +ve cocci as per the procedure of the National Mastitis Council. A 3% fresh solution of hydrogen peroxide was used for this test. A few isolated colonies which were identified according to their morphological specific characteristics were used for their emulsification in H_2_O_2_ drop. After this, the microscope slide was observed in the presence of bright light for the production of catalase in the form of small nascent Bubbles.

#### 2.5.2 Coagulase test

In this Test, Rabbit plasma was used. 1–2 drops of plasma were placed on to microscopic slide. After this, the test-heavy loop full of culture was mixed with the rabbit plasma which was taken on a microscopic slide. Incubation of this mixture was done at 37°C for a few seconds.

#### 2.5.3 Gram staining

One of the most important staining methods in microbiology is the Gram staining. Hans Christian Gram, a Danish bacteriologist, introduced it in 1882, primarily to detect pathogens that cause pneumonia [14].

#### 2.5.4 Sensitivity test

Samples positive for *S*. *aureus* were inoculated on Muller-Hinton agar in the Petri plates and antibiotic discs were placed on the surface of the inoculated agar plate. The zone of inhibition was measured through Vernier callipers and compared with the Clinical and Laboratory Standards Institute (CLSI) standard [15]. Following most frequently used antibiotics were used in this study: amoxicillin (20 μg), gentamicin (10 μg), tetracycline (10 μg), ceftiofur sodium (30μg), penicillin (10 IU), enrofloxacin (10 μg), ciprofloxacin (10 μg).

### 2.6 Experimental trial

The *in-vivo* trials in this study were organized into five groups, comprising four treatment groups and one control group (CMT negative) **(**Table 2). Five local farms with the highest incidence of mastitis cases were selected for *in-vivo* treatment trials. From each farm, five animals were selected, with one animal assigned to each treatment group.

**Table 2:** Treatment groups.

Group A	Group B	Group C	Group D	Group E
**Ceftiofur**	Streptomycin _+_ ampicillin	Enrofloxacin	Mastilep topical gel	control negative (Healthy Cows)

### 2.7 Statistical analysis

Data was analyzed by using Chi-Square, regression analysis, and Repeated measure ANOVA by SPSS (version 20.0) when *p*≤0.05).

## 3. Results

### 3.1 Prevalence and risk factors of *S*. *aureus* mastitis in dairy cows

Overall Prevalence of *S*. *aureus* mastitis in the present study was 42.5%. A total of 200 milk samples from dairy cattle were evaluated for mastitis and a percentage of *S*. *aureus* 85 (42.5%) milk samples were positive for *S*. *aureus* in district, Narowal **(**Table 3). Various recent studies also show the same result as described by [16] who designed 195 milk samples for mastitis from his study 95 samples were positive for *S*. *aureus* and the incidence of *S*. *aureus* was (46.2%), while other research [17] shows different overall prevalence of *S*. *aureus* mastitis in different region. The difference in the result of the prevalence of *S*. *aureus* mastitis might be due to the diverse husbandry managemental practices, different techniques used for diagnosis, immune status, and environmental conditions.

**Table 3:** Prevalence of *Staphylococcus aureus* mastitis in dairy cattle at Narowal.

Sampling District	Total no. of positive samples	Positive samples of *S*. *aureus*	Positive (%)	Negative samples
Narowal	200	85	42.5	115

Risk factors having significant association (*p* < 0.05) with the presence of mastitis included distance from the dung pile to the stall, frequency of dung removal per day, udder traumatic injury, floor type, teat dipping, and bedding type. Conversely, factors such as water source, lactation period, parity, milk yield, and housing type showed no significant effect (*p* > 0.05) on mastitis occurrence **(**Table 4).

**Table 4:** Risk factors.

Variable	Category / Unit	*S*. *aureus*-associated Factors		
		Total	Positive (%)	*p*-value
Distance from dung pile to stall(m)	<1	37	(25) 67.5	0.001
	1–3	68	(29) 42.6	
	>3	95	(31)32,6	
Source of water	Pipe	83	(29) 34.9	0.115
	Well	52	(23) 44.2	
	Others	65	(33) 50.7	
Dung removal per day	1	84	(18) 21.4	0.000
	2	55	(32) 58.1	
	>2	61	(35)57.3	
Lactation period (Weeks)	0–4	24	(13) 54.1	0.381
	5–9	14	(5)3.57	
	10–14	19	(9) 4.73	
	>14	21	(10)47.6	
Parity	1^st^	48	(19) 39.5	0.689
	2^nd^	46	(17) 36.9	
	3^rd^	56	(25)44.6	
	4^th^	50	(24)48	
Effect of milk yield (L)	<2	26	(11) 42.3	0.792
	2–5	75	(31) 45	
	5–10	80	(36) 36.8	
	>10	19	(7) 39.19	
Traumatic injury of udder	Yes	70	(36) 51.4	0.000
	No	130	(49) 37.6	
Housing type	Open	63	(28) 44.4	0.724
	Closed	71	(36)50.7	
	Open area	66	(21) 31.8	
Floor type	Muudy	97	(57) 46.3	0.000
	Clay	60	(34) 43.3	
	Concrete	43	(24)32.5	
Teat dipping	Yes	78	(27) 34.6	0.001
	No	122	(58) 47.5	
Bedding type	Wet dirty	78	(54) 69.2	0.000
	Dry clean	122	(31) 25.4	

### 3.2 Percentage differences of clinical and subclinical mastitis in dairy cows

Results showed that on screening with California mastitis test (CMT) 120 milk samples were positive for subclinical mastitis. Out of these 55 samples were positive for *S*. *aureus* and its overall positive percentage was 45.8%. Out of 80 clinical mastitic samples, 30 were positive for *S*. *aureus* and its positive percentage was 37.5% **(**Table 5).

**Table 5:** Percentage of clinical and sub-clinical *S*. *aureus* mastitis in dairy cattle.

Type of mastitis	Total no. of samples	Positive samples of *S*. *aureus*	Negative samples	Positive (%)
Clinical	80	30	50	37.5
Sub-clinical	120	55	65	45.8
Total	200	85	164	42.5

### 3.3 Isolation and identification and morphological/biochemical characteristics of *S*. *aureus* from mastitis milk samples

#### 3.3.1 Blood agar

**Fig 2:**
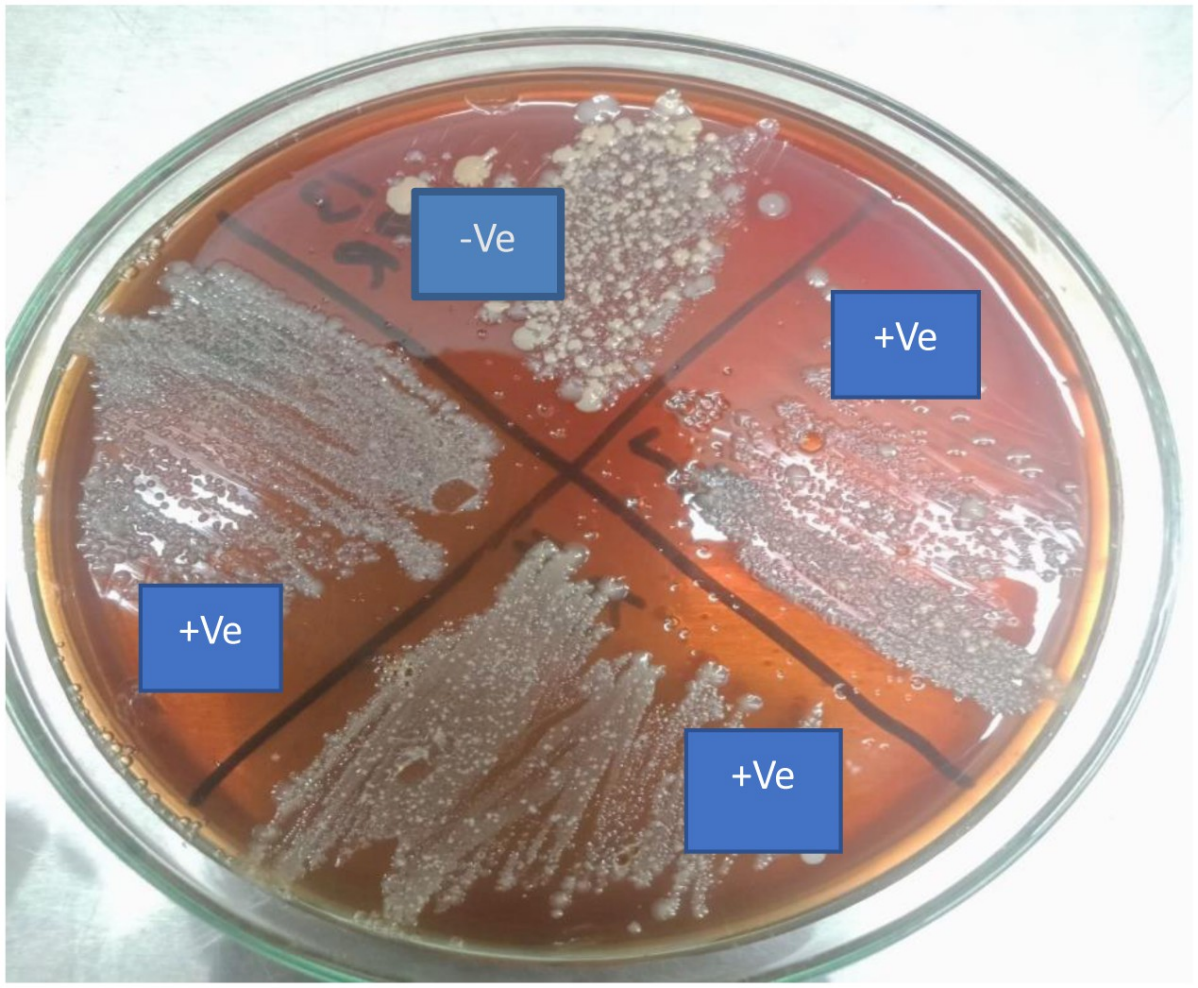
Appearance of *S*. *aureus* on blood agar.

*S*. *aureus* caused *β*-hemolysis on blood agar plates giving elevated opaque colonies with a diameter of 1–2 mm **(**Fig 2).

#### 3.3.2 Mannitol salt agar

**Fig 3:**
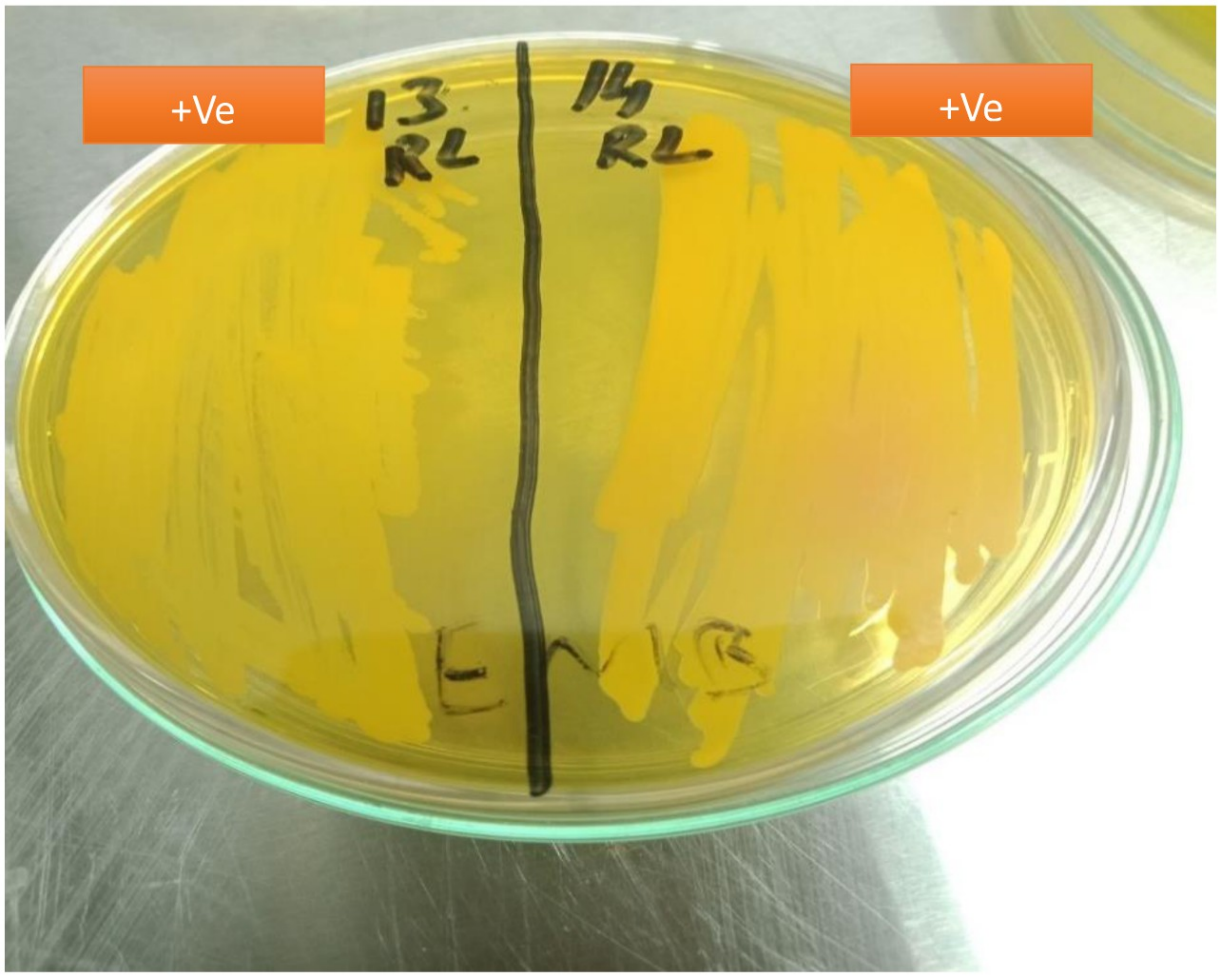
Appearance of *S*. *aureus* on MSA.

Fermentation of mannitol salt agar produced acid indicated by red color as negative for *S*. *aureus* by phenol red pH indicator and yellow as positive for *S*. *aureus* (Fig 3).

#### 3.3.3 Catalase test

**Fig 4:**
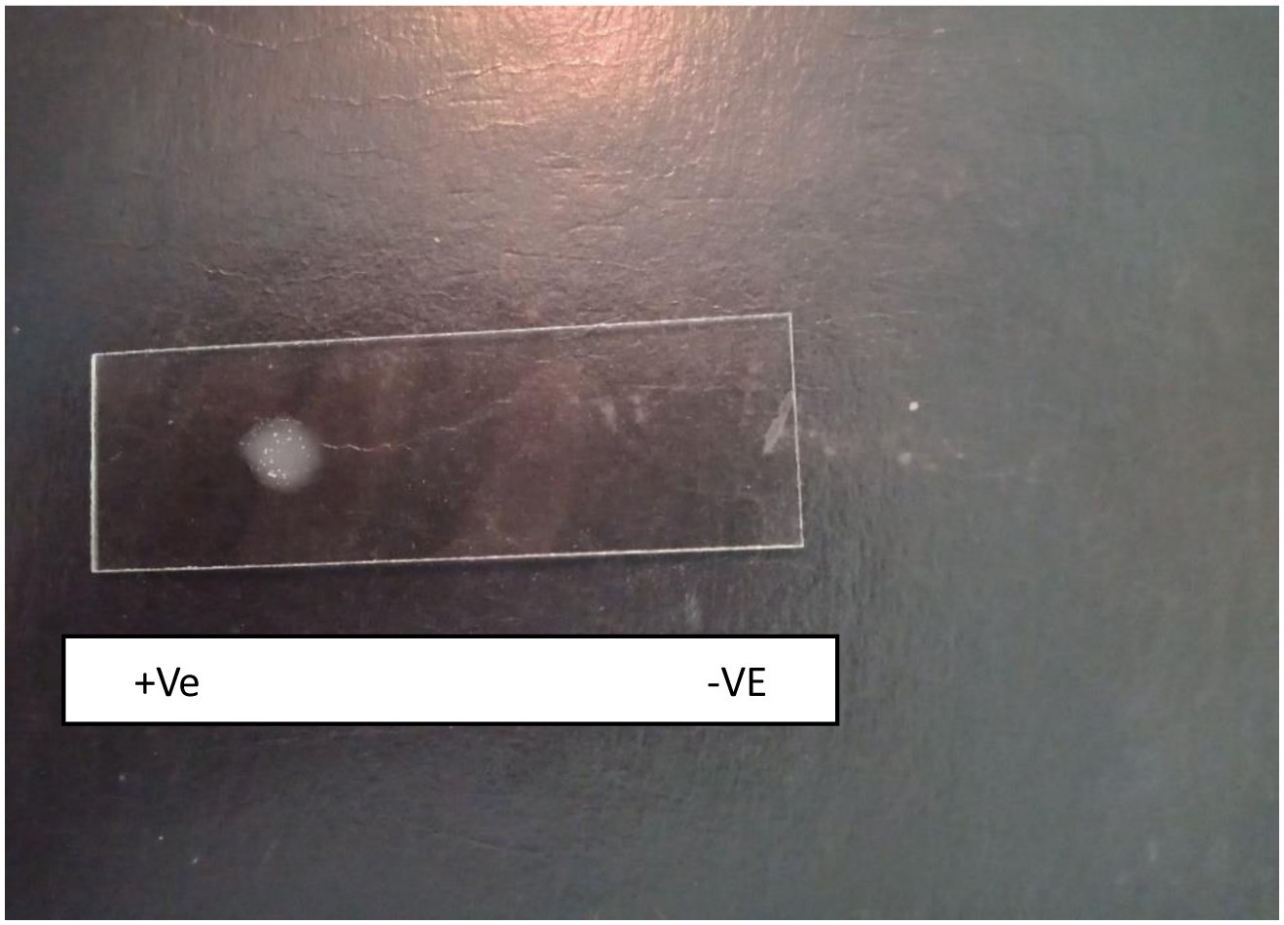
Catalase test.

**Results were interpreted as if bubbles were produced then it is positive for** *Staphylococcus***, if no bubble production, then negative for** *Staphylococcus* (Fig 4).

#### 3.3.4 Coagulase test

**Fig 5:**
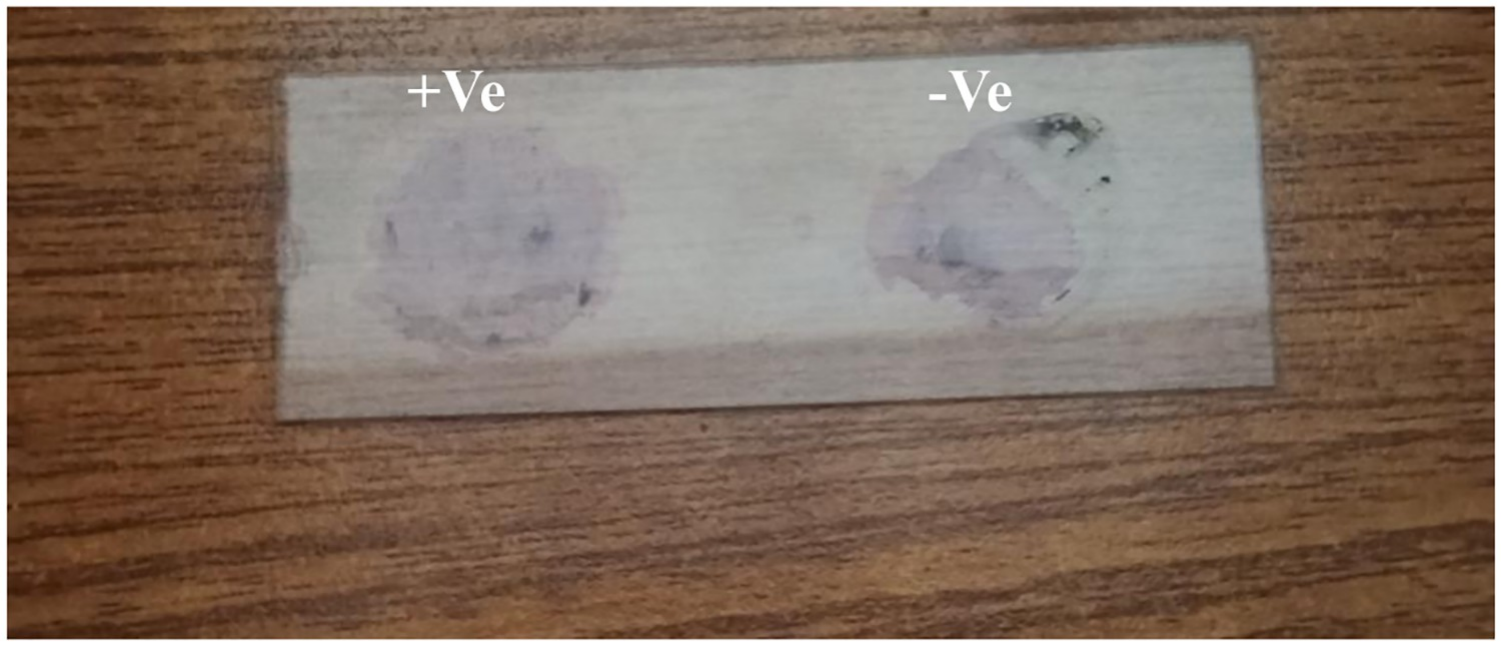
Coagulase test.

Results were interpreted as in positive there was coagulation of plasma within 10 seconds and in negative, no coagulation of plasma occurred **(**Fig 5).

### 3.4 Microscopic examination of *S*. *aureus* in mastitis milk samples

#### 3.4.1 Gram staining

**Fig 6:**
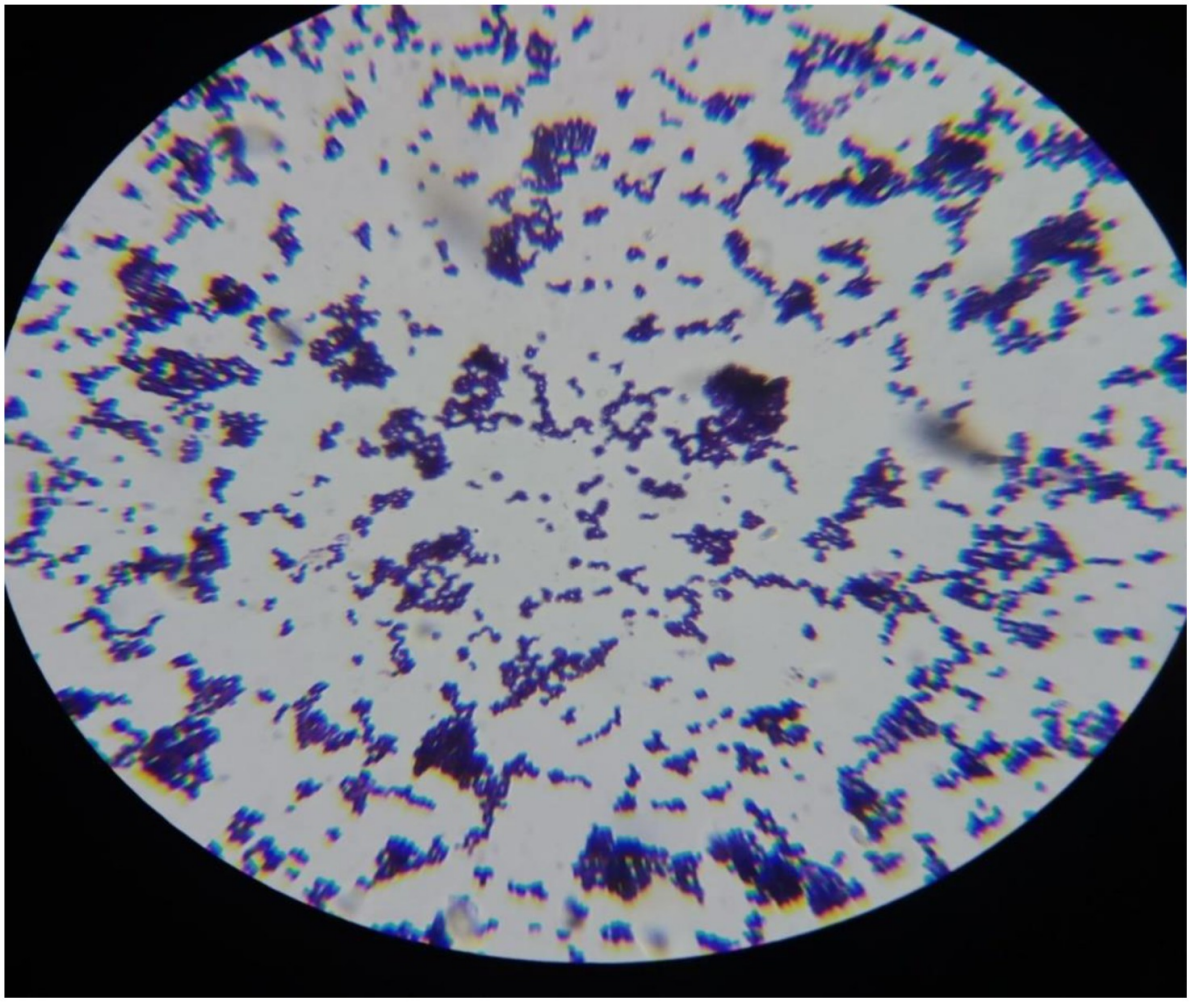
Microscopic examination of *S*. *aureus*.

Gram staining uses crystal violet or methylene blue as the main color and is frequently the first test run. Gram-positive organisms are referred to as those that preserve their original color and show up as purple-brown under a microscope (Fig 6). Gram-negative organisms that do not take up primary stain are visible as red [18].

#### 3.4.2 Procedure

Over the fixed culture, crystal violet stain is applied. The stain is poured off after 10 to 60 seconds, and any remaining stain is then washed away with water. The objective is to remove the stain while preserving the fixed culture. The smear is covered with an iodine solution for 10 to 60 seconds. This process is referred to as "fixing the dye." The slide is cleaned under running water after removing the iodine solution. Shaking off extra water from the surface. The slide is then treated with a few drops of decolorizer. Decolorizers are frequently a mixture of ethanol and acetone as a solvent. This process is referred to as "solvent treatment." In 5 seconds, water is used to rinse the slide. Stop applying decolorizer as soon as the solvent stops being colored as it flows over the slide to prevent excessive decolorization in the gram-positive cells.

### 3.5 Analysis of antibiogram of targeted isolates and antibiotic susceptibility of *S*. *aureus* isolates

#### 3.5.1 Antibiogram of isolates

**Fig 7:**
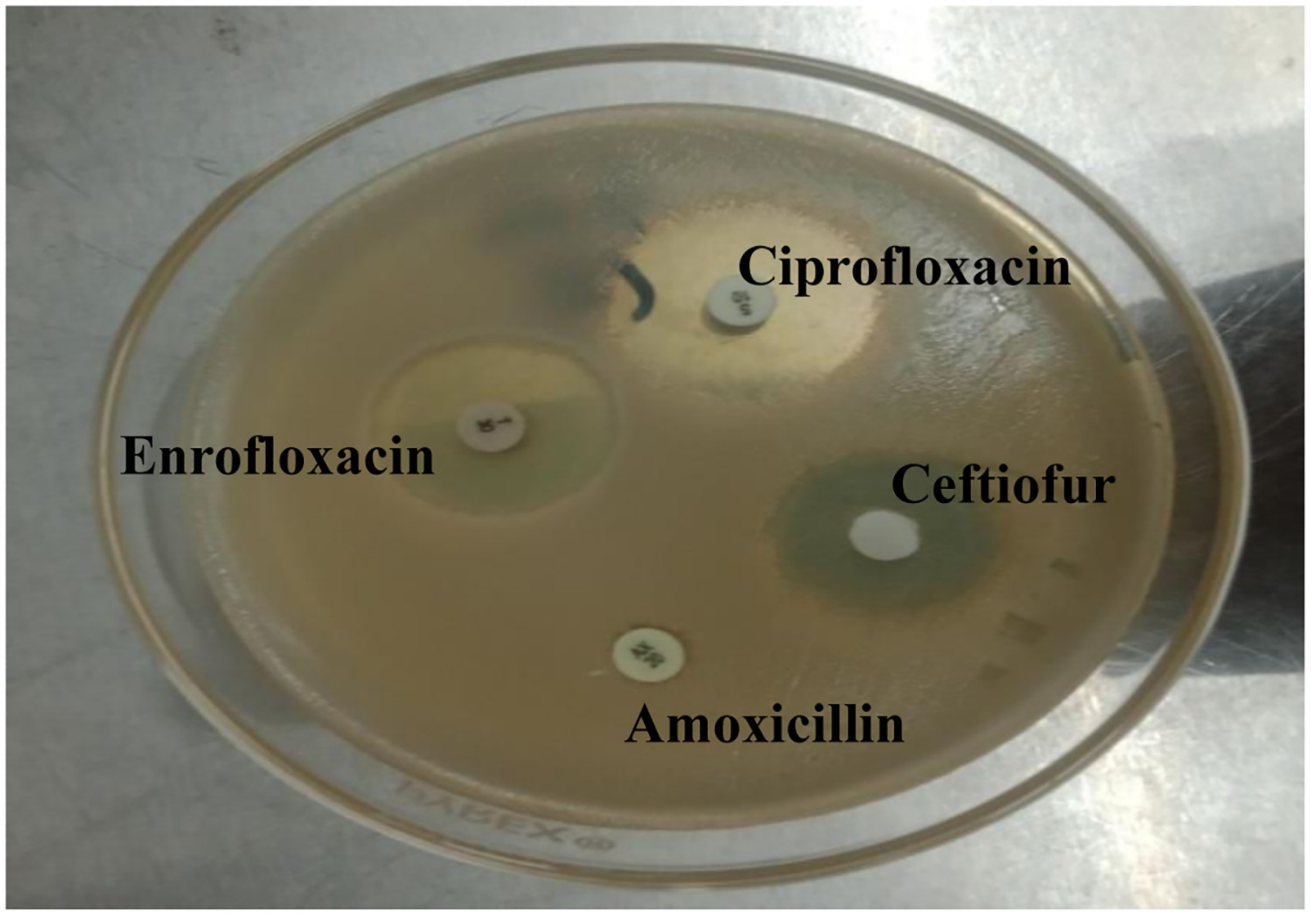
Antibiotic sensitivity zones on Muller Hinton agar.

This study revealed during isolation and identification processes that *S*. *aureus* is the most common mastitis causing pathogen. Therefore, an antibiotic sensitivity test was carried out to help in the control and better management of this distressing condition in an efficient way. An antibiogram of targeted isolates disclosed that all the isolates were highly resistant to penicillin, and amoxicillin but sensitive to Gentamycin, Ceftiofur, Tetracycline, Enrofloxacin, and Ciprofloxacin **(**Fig 7). This result was evaluated according to the CLSI standard [19] for sensitive, intermediate, and resistant zones of inhibition.

#### 3.5.2 Antibiogram of *S*. *aureus* isolates

The mean values in the table are all significant while the P-value for all antibiotics is also significant, All the *S*. *aureus* isolates were highly resistant to penicillin, and amoxicillin with mean zone of inhibition 0.00 **±** 0.00 and 0.00 **±** 0.00 mm respectively, but were sensitive to Gentamycin, Cefiofur, Tetracycline, Enrofloxacin, Ciprofloxacin having zone of inhibition 13.8750 **±** 0.63913, 13.6250 **±** 0.82240, 22.5000 **±** 0.86603, 27.6250 **±** 0.77776, 27.3750 **±** 0.67975 mm respectively **(**Table 6). The highest zone of inhibition was formed by enrofloxacin.

**Table 6:** Zone of inhibition (Mean ± Standard error).

S. No	Antibiotic	Mean ± Standard error	P-value
1	Amoxicillin	0.00 **±** 0.00	0.001
2	Gentamycin	13.8750 **±** 0.63913	0.001
3	Tetracycline	22.5000 **±** 0.86603	0.001
4	Ceftiofur	13.6250 **±** 0.82240	0.001
5	Penicillin	0.00 **±** 0.00	0.001
6	Enrofloxacin	27.6250 **±** 0.77776	0.001
7	Ciprofloxacin	27.3750 **±** 0.67975	0.001

**Fig 8:**
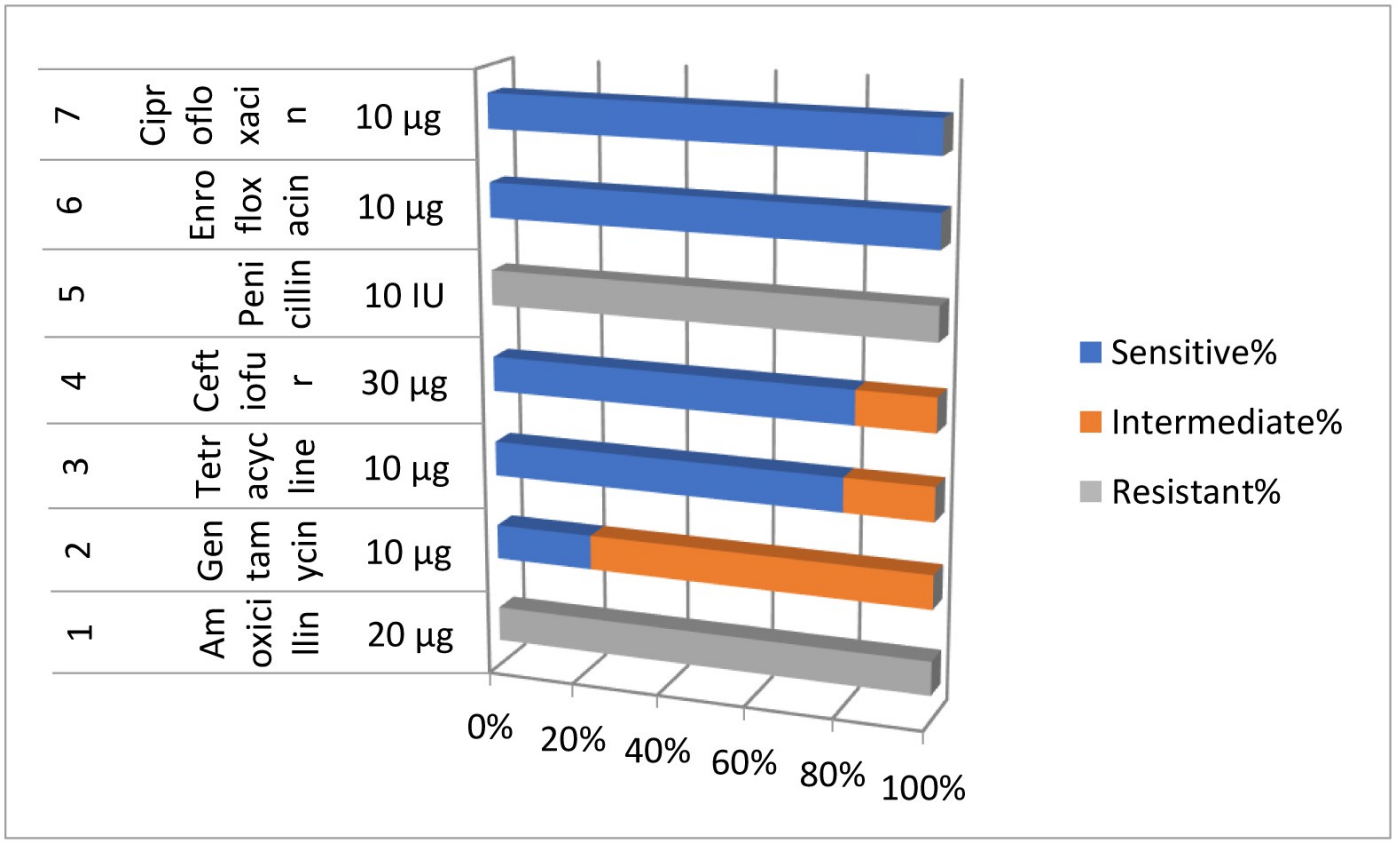
Graphical representation of different antibiotic.

Table 7 and Fig 8 reveal that Amoxicillin and penicillin showed 100% resistance to *S*. *aureus* mastitis. Similarly, Enrofloxacin, and ciprofloxacin both showed 100%. Gentamycin showed 22.35% sensitivity against *S*. *aureus*. Tetracycline showed 80% and Ceftiofur showed 82.35% sensitivity against *S*. *aureus*.

**Table 7:** Antibiotic susceptibility of *S*. *aureus* isolate.

S. No	Antibiotic	Concentration of Drugs	Sensitive%	Intermediate%	Resistant%
1	**Amoxicillin**	20 μg	0	0	100
2	**Gentamycin**	10 μg	22.35		0
3	**Tetracycline**	10 μg			
4	**Ceftiofur**	30 μg	82.35	17.64	
5	**Penicillin**	10 IU			100
6	**Enrofloxacin**	10 μg	100		
7	**Ciprofloxacin**	10 μg	100		

### 3.6 Selected antibiotics inhibited *S*. *aureus* mastitis in dairy cows

#### 3.6.1 Antibiotic trails

After Five days of treatment of groups A, B, C, and D With Ceftiofur, streptomycin plus ampicillin, Enrofloxacin, and Mastilep topical gel respectively, the efficacy of treatment was assessed through CMT and SCC at Day 5, Days 10, Day 15 and Day 30 of treatment. The Results obtained are shown in Table 8.

**Table 8:** CMT table.

Days of Experiment	Group A		Group B		Group C		Group D		Group E	
	+ ive	- ive	+ ive	- ive	+ ive	- ive	+ ive	- ive	+ ive	- ive
**0**	5	0	5	0	5	0	5	0	0	5
**5**	5	0	5	0	2	3	5	0	0	5
**10**	2	3	4	1	1	4	3	2	0	5
**15**	1	4	4	1	1	4	3	2	0	5
**30**	1	4	4	1	1	4	3	2	0	5

A = Ceftiofur B = streptomycin plus ampicillin C = Enrofloxacin D = Mastilep Topical Gel

Results revealed that on day 0, All were positive for mastitis after that CMT results showed recovery in some groups with time. The cured rate was high in the case of groups A and C and the Group E was negative control **(**Table 8).

Data on somatic cell count SCC in cows of different treatment groups (A, B, C, D and E) at various days is presented in the **(**Table 9). The SCC in all treatment groups was non significantly different at 0 day (P>0.05). On day 5, the SCC of cows significantly reduced (P<0.05) in groups A and C as compared to B and D. Whereas within the group at day 5, the SCC of cows in group A was significantly lower (P<0.05) than group B and D. Similarly group C was significantly lower (P<0.05) than group B and D, whereas at day 5, in groups B and D SCC was non-significantly different (P>0.05), likewise, the SCC of cows in groups A, C was significantly decreased as compared to group B and D at day 10. The SCC of cows of groups A and C was significantly decreased (P<0.00) at day 15 but in group B only in one animal out of 5, SCC decreased similarly in group D only in 2 animals the SCC decreased overall non-significant difference in group B and D AT DAY 15. Similarly at day 30, the SCC of cows of groups A and C was significantly decreased (P<0.05) as compared to groups B and D. A gradual decrease of SCC in groups A and C at day 5, 10, 15, and 30 but in group B the SCC count reduction was very slow at day 30 in some cases SCC was increased again. Similarly in group D, the reduction rate of SCC was very slow. The recovery rate of groups A and C was also significant, more recovery occurred from Day 5–30. In groups B and D, the recovery rate was very low. There was no significance shown in these groups.

**Table 9:** (Mean ± Standard error) for milk SCC (×10^5/^ml) in the studied groups at different days.

Groups	Days				
	0	5	10	15	30
	Mean ± Standard error	Mean ± Standard error	Mean ± Standard error	Mean ± Standard error	Mean ± Standard error
**A** **(n = 5)**	12.5600 ± 3.40705	3.6200 ± 3.26680	2.9800± 2.47022	1.8400 ±2.32873	1.1600 ±1.03102
**B** **(n = 5)**	13.4000 ± 4.72229	11.6000 ± 4.77493	10.0000 ±4.94975	9.0000 ±4.18330	8.4000 ±4.82701
**C** **(n = 5)**	14.8000 ± 3.11448	2.9400 ± 3.40118	2.3200 ±2.62240	1.7600 ±1.81742	1.3200 ±1.22352
**D** **(n = 5)**	10.2000 ± 5.01996	8.3000 ± 4.52217	6.6400 ±4.81539	5.6400 ±4.39807	4.6000 ±3.57771
**E** **(n = 5)**	0.9026± 1.23234	0.9845 ± 1.54253	0.9065 ± 1.45643	0.8901 ± 1.34455	0.8873 ± 1.35343

The Table 10 showed that a high percentage of cure rates (80%) were in groups A and C. The cured rate of group B was 20% and D was 40%.

**Table 10:** Recovery from clinical mastitis in cows of different treatment groups.

Groups	Cured	Recovery Rate%
**A**	4	80
**B**	1	20
**C**	4	80
**D**	2	40

## 4. Discussion

Percentage of clinical and subclinical mastitis Results showed that on screening with the California mastitis test (CMT), 120 samples were positive for subclinical mastitis and 80 samples were positive for clinical mastitis. Out of these 30 (37.5%) samples were positive for *S*. *aureus* from clinical mastitis samples and 55 (45.8%) samples of sub-clinical mastitis were recorded positive for *S*. *aureus* The results were significant in some studies [19]. *S*. *aureus* was isolated from clinically mastitic cattle were 39.29% While from subclinical Mastitis cattle was 80%. The result showed that the prevalence of subclinical mastitis was more than clinical in the case of *S*. *aureus* mastitis.

The parity of dairy cattle revealed that the highest percentage of *S*. *aureus* positive samples were observed during the 4th parity (48%), followed by (44.6%) in the 3rd parity, (39.5%) in the 1^st^ parity, while the lowest percentage was observed in the 2^nd^ parity (36.9%). Non-significant results were obtained as there is an association between parity and *S*. *aureus* mastitis. The chi-square analysis p-value indicated that the results (>0.05) were not in agreement with [20], the higher prevalence of disease during the first and second parity.

Effect of milk yield in causing *S*. *aureus* mastitis revealed the highest percentage of *S*. *aureus* (45%) was observed in 5–10 liters milk yield followed by 42.3% with a milk yield of <2 liters, 41.3% at 2–5 liters of milk yield and the lowest percentage of 36.8% was obtained at less than >10 liters of milk yield per day the results showed non-significant results. The p-value is no less than 0.05 as there is no significant influence of *S*. *aureus* mastitis on the average milk yield per day. Results were in agreement with [21], those who reported a negative relation between milk yield and IMI, while results were not in agreement with [22], those who reported that increased daily milk yield was a risk for IMI, who found IMI occurred more frequently in cows with higher milk production respectively.

The relationship between traumatic injury of the udder and *S*. *aureus* mastitis showed that there is an association between *S*. *aureus* mastitis due to traumatic injury of the udder. According to the findings, out of 70 mastitis-positive samples, 36 were positive for *S*. *aureus* mastitis, and they had a traumatic injury or a previous history of injury to the udder or teat. While 49 out of 130 were those animals that had no history of traumatic injury of udder. The high percentage of *S*. *aureus* mastitis in those samples that had a traumatic injury is about (51.4%). These findings of the present study are in resemblance with [23], those who reported that teat and udder injuries make bovines highly susceptible to sub-clinical mastitis.

The impact of *S*. *aureus* mastitis on the quantity of milk revealed that the Percentage of unequal quantity of milk due to *S*. *aureus* mastitis was 45.1%, while the percentage of equal quantity of milk from *S*. *aureus* mastitis was 39.5%. Results were in relation with [24], who described in his study that in subclinical mastitis, there is no visible presence of changes in the udder or milk, but milk yield decreases, bacteria are present in the secretion, and milk composition is altered.

The relationship between the *S*. *aureus* mastitis pathogen and type of housing on quantity of milk showed that the highest percentage (50.7%) of *S*. *aureus* mastitis was observed in those animals that live in closed type of housing system followed by open housing system 44.4% while lowest percentage of *S*. *aureus* mastitis was observed in open area housing system with 31.8%. Statistically, there is a no-significance association of occurrence of mastitis among different types of housing systems. The current finding was not in agreement [25] in and around Wolayta Sodo, which indicated a higher prevalence in with the findings of [26] in the Lemu Bilbilo district of Arsi Zone and intensive farms. Variability in management system reports could be attributable to differences in hygienic standards in the dairy environment and milking settings, as well as genetic disease resistance among the breeds kept in the systems, which are supported by the findings of [27].

The relationship between *S*. *aureus* mastitis pathogen and type of floor showed that the highest percentage of *S*. *aureus* mastitis (46.3%) was present in those animals that were residing on muddy floors, followed by (43.3%) on clay floors while the lowest percentage (32.5%) was obtained from those farms which had concrete floor Chi-square analysis’s p-value indicated that the results were significant is less than 0.05. The results were not in agreement [28]. The greatest incidences of mastitis occurred when animals were maintained on a brick floor, according to data on the prevalence of mastitis as a function of floor type (brick, cemented, and Muddy). A clean, Muddy floor, cemented floor, and appropriate sleeping material may help to solve the problem. The findings countered those of [29], who claimed that an unclean floor, brick floor, and hard bedding could all be predisposing factors for mastitis in pri-urban locations.

The Relationship between *S*. *aureus* mastitis pathogen and dipping revealed that those sheds that had no history of pre and post-dipping showed (47.5%) of *S*. *aureus* mastitis while those with a history of dipping showed (34.6%) results. Statistically, there is a significant association between teat dipping and the occurrence of mastitis. The results are significant as obtained by chi-square analysis because the p-value is less than 0.05. At the farm level, dipping was mostly not performed, which has a high chance of sub-clinical mastitis. These results are in line with the findings of [30].

Prevalence of *S*. *aureus* mastitis in dairy cattle at quarter levels showed that Quarter-wise highest positive percentage of *S*. *aureus* mastitic pathogen was obtained in Right rear (RR 51.61%) followed by right front (RF 39.5%), left rear (LR 38.8%) while least percentage was obtained in left front (LF 36.1%). The table showed nonsignificant results. The Higher percentage of infection in the hind quarter might be due to unhygienic hind legs, and incomplete milk left in the quarter favors the growth of bacteria, which may cause damage to the teats and udder membrane [31].

Type of bedding showed that the highest percentage of *S*. *aureus* mastitis (69.2%) was present in those animals that were residing in Wet, dirty bedding while the lowest percentage (25.4%) was obtained from those farms that had Dry clean bedding. Statistically, there is a significant association of the occurrence of mastitis among different types of bedding. Results were in relation with [32].

Distance from Dung pile to stall revealed that the highest percentage of *S*. *aureus* mastitis (67.5%) was present in those animals in which a distance from dung pile to stall was less than <1m, followed by (42.6%) in those animals which have distance from dung pile to stall was 1–3 m while least percentage (32.6%) was obtained from those animals which have distance from dung pile to stall was >3 m. Statistically, there is a significant association of study with [33] the occurrence of mastitis at a distance from a dung pile to a stall.

Source of water results indicated that the prevalence of *S*. *aureus* mastitis was recorded as high in other sources of water (50.7%), followed by wells (44.2%) and pipe systems (34.9%). Statistically, there is a non-significant association of occurrence of mastitis among Sources of water. Results were about [34] those who described drinking water sources other than public water as associated with an increased rate of mastitis. The relationship between the source of drinking water and environmental mastitis was similar.

Dung removal per day results showed the highest percentage of *S*. *aureus* mastitis (58.1%) were present in those farms where dung was removed after 2 days, followed by (57.3%) almost the same where dung was removed after more than 2 days. At the same time, the lowest percentage (21.4%) was obtained from those farms where dung was removed daily. Statistically, there is a significant association between the occurrence of mastitis and dung removals per day. Results from [35], who described the survey regarding manure removal the majority of dairy farms remove their manure weekly, where the chances of mastitis were 98%, and some remove their manure daily, where chances of mastitis were 2%.

Lactation period results showed Lactation period wise Result showed Lactation period wise highest positive percentage of *S*. *aureus* mastitic were obtained from 10–14 weeks of lactation (54.71%), followed by (51.02%) obtained from more than 5–9 weeks, and (41.86%) obtained from 10–14 weeks while least percentage was obtained from 0–4 weeks (23.63%) showed No significant results. Mastitis risk increased as lactation progressed through its various stages, as described previously [36]. However, several other studies indicated that the prevalence of mastitis was higher in the early stage of lactation [37]. The reason for the higher incidence of mastitis at a later stage of lactation might be due to the continuous milking of lactating animals for a long period, exerting negative pressure and causing damage to the epithelial lining of teats.

This study revealed during isolation and identification processes that *S*. *aureus* is the most common mastitis-causing pathogen. Therefore, an antibiotic sensitivity test was carried out to help in the control and better management of this distressing condition in an efficient way. An antibiogram of targeted isolates disclosed that all the isolates were highly resistant to penicillin and amoxicillin but sensitive to Gentamycin, Ceftiofur, Tetracycline, Enrofloxacin, and Ciprofloxacin. Results showed a significant relation as described by [38], Penicillin and amoxicillin were highly resistant to *S*. *aureus* mastitis. Similarly, Gentamycin, Ceftiofur, Tetracycline, Enrofloxacin, and Ciprofloxacin were sensitive to *S*. *aureus* mastitis. Results were in relation to [39]. *S*. *aureus* isolates were resistant to tetracycline, enrofloxacin, gentamicin, and enrofloxacine. The highest efficacy was Enrofloxacin, which was by the studies [40] that reported that most bacterial strains isolated from mastitis milk samples were highly sensitive to enrofloxacin, similar results were observed [41].

Mastitis is caused by numerous microbial agents that gain entry into the teat canal of the udder and multiply in the parenchyma of the mammary gland, but *S*. *aureus* is the most prevalent and important, it responds poorly to treatment with antibiotics. In the present study, a higher prevalence of this microorganism was reported, which resembles the findings of higher prevalence previously described by [42, 43]. The higher prevalence of *S*. *aureus* is ascribed to the whole distribution of microorganisms on the skin of the teat and udder and inside the mammary gland [44]. The study was intended to evaluate the efficacy of different treatments against *S*. *aureus* clinical mastitis in lactating cattle to cure the infection. For this purpose, 20 lactating cattle affected with clinical mastitis were selected and divided into 4 groups: A, B, C, and D having 5 numbers of animals each. Group A was treated with Cefent DC (Ceftiofur Hydrochloride) at a dose of 500mg/10ml intra-mammary, group B with streptomycin at dose rate 11mg/kg, and ampilicine with dose rate 22mg/kg were used in combination and was injected intra-mammary, group-C with Enroject (Enrofloxacin) 300mg/10ml intra-mammary and group-D was treated by Mastilep topical herbal gel was a tube of 125mg (Eucalyptus globulus + Curcuma longa) for 5 days consecutively twice a day. The overall Cure rate of Groups A, B, C, and D was 80%,20%,80%, and 40% respectively. In group A ceftiofur, whose cure rate was 80% after 30 days, Results showed a significant relation as described by [45], who described the Efficacy of ceftiofur therapy against all clinical mastitis was 38.8%, 53.7%, and 65.8% for the 2, 5, and 8-day ceftiofur treatment. The efficacy of the drug increases day by day. In group B, streptomycin plus ampicillin, both used in combination, showed a cure rate of 20% because the streptomycin was least effective against *S*. *aureus* mastitis, The Result showed a significant relation as described by [46]. In Group C Enrofloxacin, whose cure rate was 80%, the Result significantly showed a relation as described by Cure rates on day 21 after treatment were 75%, Uniquely high milk enrofloxacin concentrations were obtained after intramammary administration of enrofloxacin. In group D, Mastilep topical gel showed a cure rate of 40%, significantly related as described by [47].

## 5. Conclusion

Several risk factors were identified in the study that have previously been reported as risk factors in other research. These risk factors are mostly responsible for the increased risk of *S*. *aureus* mastitis. According to the research, controlling these variables may help to reduce the risk of *S*. *aureus* mastitis in cattle from commercial and sustainable dairy forms in Pakistan and other Asian developing countries. Based on the findings of this study, it is recommended that managemental control and biosecurity measures be given priority attention. Proper management and strict bio-security practices can help control and prevent the spread of *S*. *aureus* mastitis. The research highlights a prevalence of *Staphylococcus* aureus mastitis in the Narowal, Zafarwal, and Shakargarh, also experiencing notable infection rates. This emphasizes the urgent need for targeted interventions and improved herd management practices to curb the spread of mastitis in these regions. The in-vitro analysis indicates that ciprofloxacin and enrofloxacin exhibit greater sensitivity compared to other antibiotics like amoxicillin, gentamicin, tetracycline, penicillin, and ceftiofur. Penicillin and amoxicillin showed complete resistance, signifying limitations in their efficacy against *Staphylococcus* aureus mastitis. The in-vivo findings provide valuable insights into treatment approaches. Among the tested interventions, intramammary tube administration of enrofloxacin and ceftiofur demonstrated higher effectiveness in managing *Staphylococcus* aureus mastitis compared to Mastilep topical gel and streptomycin + ampicillin combination.
